# What matters to patients following total knee arthroplasty? A grounded theory of adapting to a knee replacement

**DOI:** 10.1186/s12891-022-05695-x

**Published:** 2022-09-06

**Authors:** Ellen Randall, Stirling Bryan, Charlyn Black, Laurie J. Goldsmith

**Affiliations:** 1 School of Population and Public Health, University of British Columbia, 2206 East Mall, Vancouver, BC V6T 1Z3 Canada; 2 Centre for Clinical Epidemiology and Evaluation, Vancouver, Canada; 3 Centre for Health Services and Policy Research, Vancouver, Canada; 4 Faculty of Health Sciences, Simon Fraser University, 8888 University Drive, Burnaby, BC V5A 1S6 Canada; 5 GoldQual Consulting, Toronto, Canada

**Keywords:** Total knee arthroplasty, Adapting, Patient satisfaction, Patient experience, Qualitative research, Grounded theory

## Abstract

**Background:**

Globally the volume of total knee arthroplasty (TKA) is on the rise, reflecting aging populations, an associated increase in treatment of osteoarthritis, and a desire for improved quality of life. There is evidence that as high as 15 to 20% of patients are not satisfied with their TKA results and efforts need to be made to improve these rates. This study set out to identify what patients consider important when reflecting on TKA satisfaction, to pave the way to identifying service transformation opportunities that will enhance patient-centred care and satisfaction with this procedure.

**Methods:**

Twenty-seven TKA recipients were recruited in the province of British Columbia, Canada. Semi-structured interviews were conducted about participants’ experience and satisfaction with TKA, three to four years post-surgery. Grounded theory was employed to analyze participants’ stories about what was front of mind when they reflected on satisfaction with their new knee.

**Results:**

Participants described their post-TKA knee in terms its *adequacy*: how it felt and worked, and how it matched their pre-surgical expectations. The central element of their stories was the process of *adapting,* which gave rise to their perceptions of adequacy. Adapting comprises the patient experience of physically integrating and cognitively accepting their new knee. Patterns of adapting reflect the level of the new knee’s achieved adequacy and the straightforwardness of the adapting process.

**Discussion:**

The conceptualization of adequacy and the process of adapting allow a patient-centred understanding of what patients experience following TKA. For participants who did not readily achieve the adequacy they had anticipated, the challenges they experienced during adapting dominated their stories. Participants’ adapting stories afford key insights into how the health care system could adjust to better support TKA patients, and improve rates of satisfaction with this procedure.

**Conclusions:**

The process of adapting lends itself to system intervention in support of enhanced post-TKA outcomes and satisfaction. These interventions could include the development of a care model including long-term clinical support for patients whose knees do not achieve desired results on schedule, and collaborating with patients to set and manage reasonable expectations about how their post-TKA knee will feel and function.

## Background

Primary total knee arthroplasty (TKA) is performed to relieve symptoms typically associated with degenerative osteoarthritis [1–3]. Globally the volume of this procedure is on the rise [1, 2, 4–7], and it is the most frequently performed type of joint replacement surgery in Canada [7] and the United States [2, 8]. The rise in surgical volume reflects an aging population and an associated increase in the diagnosis and treatment of osteoarthritis [9], as well as a desire for improved quality of life [2]. Studies from a variety of countries, however, have found that as high as 15 to 20% of patients are not satisfied with their TKA results [10–14]. Given the trend towards increased volume, and the high direct costs of this procedure [7, 15], efforts need to be made to improve satisfaction rates and enhance return on this investment. To that end, with a growing emphasis on patient-centred care [16–19] and recognition of the importance of incorporating patients’ voices in the evaluation and improvement of health care delivery [5, 20, 21], health policymakers, clinicians, and researchers need to understand better what patients value when considering satisfaction with TKA.

Defined by Donabedian as a “judgement on the quality of care in all its aspects” ([22], p. 1746), patient satisfaction is widely recognized as a key health care outcome and important quality indicator [5, 23, 24]. Despite its importance, patient satisfaction remains a concept that defies a single, coherent, agreed upon definition [25–27]. It has, nonetheless, been much measured [25, 27]. Research into patient satisfaction following TKA reflects this emphasis on measurement, tending to focus on identifying correlates of satisfaction rather than defining what satisfaction means for a TKA patient. Described correlates for post-TKA satisfaction include pain relief, improved physical functioning, and met expectations [11, 13, 14, 28–31]. Despite this work on identifying correlates of satisfaction, there remains a sense that we do not yet sufficiently understand the mechanics of post-TKA satisfaction to effectively address dissatisfaction, and there continues to be a call to better this understanding [14, 32]. Learning directly from patients what satisfaction means to them, and what they prioritize when considering satisfaction, remains under-studied and is a critical input for a more comprehensive understanding of post-TKA satisfaction to support system-based efforts to assess and improve post-TKA satisfaction rates.

Currently, most studies in the literature investigate post-TKA satisfaction in the first year following surgery [33]. There is a need to look beyond that timeframe, given knee implants that are designed to deliver long-term benefits [34] and the variation in patients’ recovery times [35–37]. Exploring how TKA recipients think about satisfaction well beyond that first year, after they have had considerable time to potentially resolve lingering post-surgical issues and fully assimilate their post-surgical knee into their daily lives, provides important additional perspective to the knowledge base.

As part of a broader research program examining patient satisfaction with TKA [31, 38], this qualitative study set out to address these knowledge gaps by learning directly from patients what they consider when they think about satisfaction with TKA three to four years post-surgery. Applying a qualitative lens to a complex phenomenon such as patient satisfaction allows for a broad and open exploration into patients’ perspectives, shedding light on aspects of the phenomenon that quantitative research may not reach [39]. The objective of this study was to identify what patients consider important when asked to reflect on long-term satisfaction with TKA in order to enrich our understanding of what affects satisfaction with this procedure over time. Gaining a nuanced, firsthand understanding of what matters most to TKA recipients when evaluating their new knee will pave the way to identifying service transformation opportunities that will enhance patient-centred TKA care.

## Methods

To support our objective of learning what is front of mind for TKA recipients when considering satisfaction, we employed grounded theory to analyze participants’ personal narratives relating to satisfaction with TKA three to four years post-surgery. The signature methods of grounded theory, including theoretical sampling, concurrent data collection and analysis, and constant comparison, are well-suited to exploring a complex phenomenon like patient satisfaction because they enable the identification of the key processes underlying the phenomenon being studied [40].

### Study sample

Participants for this qualitative study were recruited from the 3-year cohort of the provincial *Patient Experience with Arthroplasty of the Knee* (PEAK) cohort [31] in the province of British Columbia (BC). The original PEAK cohort consists of 515 adult patients (age ≥ 19 years) from across BC, who had primary TKA surgery in 2012–2013. The PEAK 3-year cohort (*n* = 314) comprised PEAK cohort members who completed the *PEAK 36-month Post-Surgery Questionnaire* mailed out on the third anniversary of their primary TKA surgery, gathering data on outcomes and patient satisfaction. Members of the PEAK 3-year cohort who indicated a willingness to participate in further studies (*n* = 236) were eligible for recruitment for this qualitative study.

The target sample size for our study was 30 participants. It was anticipated that 30 interviews would provide sufficient “information power” [41] and allow for maximum variation on key characteristics [42] such as sex; age; geographic representation; and self-reported measures from the *PEAK 36-month Post-Surgery Questionnaire* including satisfaction, pain, and pre-surgical expectations. A sample of 30 interviews also falls within the recommended sample size range for grounded theory [43–45].

Initial purposeful sampling was based on findings from earlier qualitative and quantitative studies with the full PEAK cohort [31, 38], with a particular focus on aspects such as self-reported pain levels and social support. To gain as broad an exposure as possible to those who were not satisfied with their new knee, initial sampling also focused on two satisfaction-related questions from the questionnaire. Respondents who reported being overall “very dissatisfied,” “dissatisfied,” or “neutral” with their results were over-sampled, as were those who indicated “no” or “unsure” in response to the survey question that asked if they would have had the surgery if they had known in advance what their actual results were going to be.

As concurrent data collection and analysis unfolded, and early categories were identified in the data, sampling progressed to theoretical sampling. Additional details from the *PEAK 36-month Post-Surgery Questionnaire* were added as sampling criteria to ensure representation for emergent analytic categories (e.g., ease with usual activities, insurance for physiotherapy and supplemental health services) [40]. Closure of recruitment was based on an assessment of saturation as concurrent analysis showed that data from the later interviews were not adding critical new information [46].

Potential participants were approached by letter asking if they would be willing to take part in an in-depth interview and outlining how this interview study added to the PEAK research project in which they were already participants. These letters were then followed by a phone call from the lead author [ER], during which she introduced herself as the interviewer for this follow-up study and described her ongoing role on the PEAK team. People who expressed interest in participating were provided with the consent form for review. After consent was attained, study participants were scheduled for their interviews.

### Data collection

The initial semi-structured interview guide included questions on a range of potential influences, including initial expectations and the extent to which they were met, symptom relief, quality of care, personal support, and the effect of their knee replacement on their day-to-day life. These questions were developed based on a review of both the TKA literature and findings from earlier PEAK studies [31, 38]. Over the course of concurrent data collection and analysis, the interview guide evolved with questions added, refined, or dropped based on the emerging analysis [40]. For example, questions about personal support were reduced because participants were more interested in and focused on perceived system support, and a closing question was added to explore the alignment of a patient’s described sense of satisfaction and their sense of whether having the TKA was still the right thing to have done.

Interviews were scheduled in the six-month period following a participant’s completion of the *PEAK 36-month Post-Surgery Questionnaire*. Interviews were conducted by a single interviewer [ER], took place at interviewees’ homes, and averaged 45 min in length. The interviewer works professionally as a qualitative interviewer, and was one of the interviewers for the original PEAK study. For the majority of interviews, only the participants were present; in a handful of instances, participants’ spouses were present but did not participate. At the end of each interview, the participant was paid a cash honorarium. Interviews were audio-recorded and transcribed verbatim for analysis. After each interview, the interviewer wrote an interview debrief, noting unusual or compelling data and whether the interview data supported or contradicted emergent findings in the early analysis. Throughout the interview period, regular debrief sessions were held by three of the paper co-authors [ER, LJG, SB].

### Data analysis

Analysis began following the first interview and continued throughout data collection. Coding was undertaken in stages using the qualitative software NVivo 12 (QSR International Pty. Ltd). Initial coding identified preliminary codes, with a focus on dimensions relating to satisfaction that were elemental to the participants’ stories. Focused coding then prioritized key conceptual codes and synthesized data into exploratory categories and sub-categories [40]. Theoretical coding affirmed and refined categories and we mapped the relationships between categories into a theory representing the stories we saw in the data [40]. Constant comparison was used through every stage of coding to uncover the characteristics of emergent categories [40]; it also provided a means of checking the fit of the entire dataset with these categories [46]. To affirm the theory that emerged from these coding stages, we also employed axial coding to verify and map relationships between categories and sub-categories [40]. Using Strauss and Corbin’s coding paradigm, this complementary exercise allowed the identification of a central category [46]. This central category, capturing the principal process of interest, was coded using a gerund label. The gerund form signifies the process’s active role in the theory and keeps the theoretical lens focused on actions rather than static events or concepts [40]. Actions also provide the best opportunities for intervention. Analysis was primarily undertaken by the lead author [ER], advanced by regular discussions with two co-authors [LJG, SB]. The remaining co-author [CB] provided occasional checks on the face validity of the analysis. Incorporating multiple reviews of the analysis by co-authors supported analytic rigour [47].

## Results

Twenty-seven people were interviewed. With the exception of one person, all who were approached about participating in the study were willing to participate. The majority of participants were female, self-identified as “North American”, and were married or living common-law. For most, annual household income was less than $60,000 and completed education was either at the high school or college/technical school level. As planned, well over half the participants reported not being satisfied with their TKA (see Table 1).

**Table 1:** Descriptive characteristics for interview participants (*n* = 27)

**Participant Characteristics**		
Age (years)	Mean: 71 Min: 54 Max: 96	
		**Count (%)**
Sex	Female	18 (67)
	Male	9 (33)
Marital Status	Married / Common-law	19 (70)
	Widowed	3 (11)
	Divorced	5 (19)
Annual household Income	< $40,000	10 (37)
	$40,000 to < $60,000	5 (19)
	$60,000 to < $80,000	2 (7)
	> $80,000	8 (30)
	Missing	2 (7)
Education	< High School	1 (4)
	High School Graduate	8 (30)
	College/Technical School Grad	7 (26)
	University Undergrad Degree	2 (7)
	University Graduate Degree	5 (19)
	Other	2 (7)
	Missing	2 (7)
Ethnicity	North American	17 (63)
	European	5 (19)
	Pacific Asian	2 (7)
	Aboriginal (First Nation)	1 (4)
	Missing	2 (7)
Provincial health region	1	6 (22)
	2	3 (11)
	3	6 (22)
	4	5 (19)
	5	7 (26)
Overall satisfaction with results (at 36 months)^a^	Very Dissatisfied	6 (22)
	Dissatisfied	10 (37)
	Neutral	6 (22)
	Satisfied	2 (7)
	Very Satisfied	3 (11)
Willing to have TKA again, knowing what they now know (at 36 months)^b^	Yes	13 (48)
	No	8 (30)
	Uncertain	6 (22)

^a^ PEAK 36-month questionnaire question: Overall, how satisfied are you with the results of your knee replacement surgery?

^b^ PEAK 36-month questionnaire question: Finally, knowing what your knee replacement did for you, if you could go back in time, would you still have undergone this surgery?

### Shifting the focus from satisfaction to the concept of adequacy

When asked what satisfaction with TKA meant to them, participants tended to engage somewhat cursorily with the actual concept. Their primary interest was not in assessing a value for their satisfaction. Their stories, rather, centred on the perceived *adequacy* of how their post-TKA knee felt and worked. Adequacy was an assessment of how well a person’s symptom resolution and resumption of function met their expectations about how their new knee would feel and work following surgery. The more a new knee felt and worked the way a person hoped it would, the better they found its adequacy to be. The better that perceived adequacy, the more positive a person was about their new knee.

### Understanding the process that determines the adequacy of a post-TKA knee: the theory of adapting

**Fig. 1:**
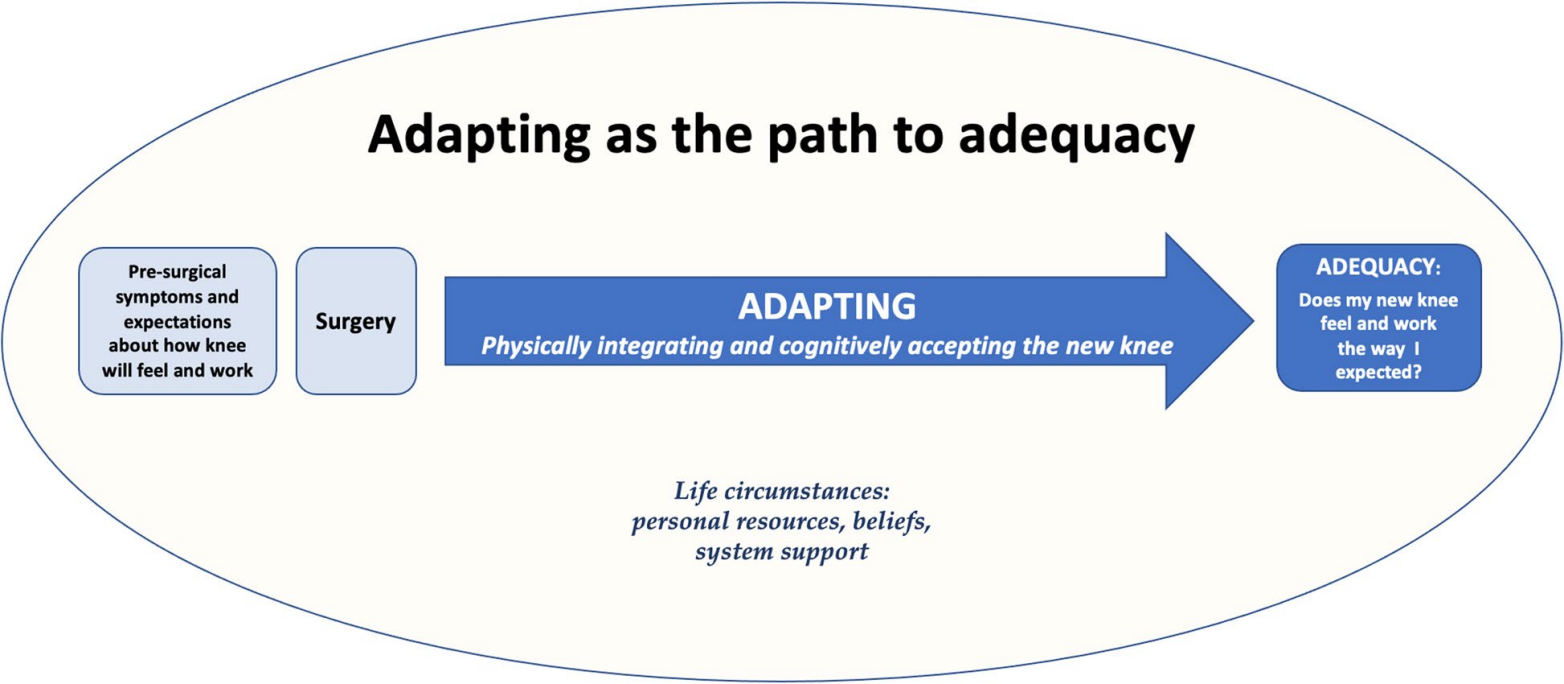
The process of adapting following total knee replacement

Our theoretical analysis identified that the adequacy of a post-TKA knee is determined through the active process of ***adapting*** (Fig. 1). Adapting is a person’s lived experience of physically and cognitively adjusting to their post-TKA knee. It comprises two interdependent sub-processes—*integrating* and *accepting*—which unfold as a person heals from their surgery*.*

#### The integrating sub-process

Integrating is the active *physical* labour of adapting. This sub-process encompasses the activities undertaken by a person that support their physical adjustment to their new knee. The purpose of integrating is to move a person through recovery to the best possible symptom resolution and resumption of function, enabling easy incorporation of the new knee into body and life. Integrating begins following surgery, acting with the body’s natural ability to heal and acting on the physical changes produced by the TKA.

When integrating is straightforward, it includes only the routine activities of physical recovery, such as attending prescribed physiotherapy sessions. If integrating requires extra effort, it can also include activities to improve symptoms that are slow to resolve (e.g., additional physiotherapy or alternative therapies such as massage); adjustments to accommodate persistent symptoms (e.g., modifications to one’s environment such as moving items from lower cabinets to higher cabinets to avoid bending); and responses to newly emerging symptoms (e.g., further diagnostics).

#### The accepting sub-process

Accepting is the *cognitive* labour of adapting. This sub-process encompasses the activities undertaken by a person that support their emotional adjustment to their new knee. These activities include monitoring and evaluating symptom resolution and the new knee’s ability to function. The purpose of accepting is to assess the adequacy of the new knee’s fit with a person’s expectations about how the knee should feel and work following a TKA.

The monitoring and evaluating activities of accepting begin once a person starts to expect improvements in their post-TKA knee, after initial healing and rehabilitation are finished. These activities are not inherently structured with regular check-in times. Point-in-time assessments of adequacy are typically prompted by an external trigger, such as reaching a key milestone or having someone enquire about how the new knee is doing. During accepting, a person considers questions such as: Is the knee improved enough to recede into the background or does it require ongoing attention? Does the knee allow me to do enough of what I hoped to do, or are those hopes as yet unmet?

When accepting is straightforward, it consists of a series of basic assessments to gauge adequacy and whether the new knee is now good enough. When there are delays or setbacks in the way a new knee meets a person’s expectations, accepting involves decision making about whether to make extra efforts to improve symptoms that have not fully resolved, or to address newly emerging symptoms. Decision making may also include decisions about if and how to revise expectations to accommodate persistent symptoms and/or new demands on the knee.

#### How integrating and accepting work together in the process of adapting

As the activities of integrating unfold, and symptom resolution and functionality start to become apparent, a person begins to use the sub-process of accepting to assess the new knee’s adequacy. These assessments trigger decisions about need for further integrating efforts to resolve symptoms. Through this decision making, accepting directs integrating and determines its duration, intensity, and activities. Integrating continues until symptoms are fully resolved and function restored, or if this ideal state proves unattainable, until these activities are deemed to be of no further benefit. When a new knee’s hoped-for state proves unattainable, in addition to deciding about the value of further integrating, a person’s accepting sub-process may also involve decision making about downgrading their original expectations. As the arbiter of adequacy, accepting decides when a knee has attained sufficient adequacy for that individual, whether that is optimal adequacy or the knee is simply good enough. Once a person feels that an optimal or good level of adequacy has been achieved, or that their new knee is at least tolerable and further efforts won’t improve it, the sub-processes of integrating and accepting become inactive.

#### Variation in the process of adapting

The process of adapting varies first and foremost in people’s levels of achieved adequacy—the extent to which their new knee feels and works as they expected it would. Levels of adequacy range in value from optimal adequacy to insufficient adequacy.

Adapting also varies in the degree to which it is a straightforward process. Adapting is straightforward when symptom resolution and resumption of function require only the time and effort one would anticipate following a major surgery. Adapting that is not straightforward makes demands on both the integrating and accepting sub-processes. Delays and failures in symptom resolution and resumption of function typically necessitate more integrating activity. Prolonged integrating requires more monitoring and evaluation, so accepting is also more labour-intensive and protracted.

Lastly, adapting varies in its state of activity at a point in time—i.e., whether it is active or inactive. Adapting is inherently open-ended. As long as a person is working towards improvements in how the new knee feels and works, adapting remains active. When a person feels that the post-TKA knee has achieved a state of adequacy they feel is good enough, and/or they deem that further efforts at integrating are unlikely to render improvements, the process of adapting becomes inactive. This inactive state can change in response to changing conditions. A person can move back into adapting if new symptoms or issues with the prosthesis or new demands on the knee arise.

### Patterns of adapting to the post-TKA knee

The three sources of variation described above (adequacy, straightforwardness, state of activity) combined in five patterns of adapting, anchored by participants’ achieved adequacy (Table 2).

**Table 2:** Patterns of adapting in the study sample at three to four years post-surgery

Adapting pattern	Level of new knee’s adequacy	Straightforwardness (amount of extra effort required)	State of adapting
**I**	Optimal adequacy	Straightforward (no extra effort)	Inactive
**II**	Good adequacy	Not straightforward (minimal extra effort)	Inactive
**III**	Tolerable adequacy	Not straightforward (considerable extra effort)	Inactive
**IV**	Insufficient adequacy	Not straightforward (concerted and ongoing extra effort)	Active
**V**	Tolerable adequacy by default (knee deprioritized due to life circumstances)	Straightforward by default (curtailed effort due to life circumstances)	Inactive

Four of these patterns formed a rough continuum with regard to adequacy and straightforwardness; the fifth pattern was an outlier. Across the first four patterns, higher assessments of adequacy at the time of the interview were typically accompanied by descriptions of greater straightforwardness in adapting. People in Pattern I, whose adequacy was optimal, described straightforward adapting. Those in Patterns II through IV, whose knees were perceived as having less than optimal adequacy, described adapting that was increasingly not straightforward, with delays or setbacks in symptom resolution and resumption of function which affected the time and energy required for both integrating and accepting. The exception to this relationship between adequacy and straightforwardness was found in the stories of the people in Pattern V. These individuals achieved tolerable adequacy by default as significant life events had occurred close in time to surgery for these people, pre-empting and foreshortening their process of adapting.

The patterns also varied in the state of adapting at the time of the study. Adapting was inactive for participants who felt their knee was adequate enough, and active for those who felt their knee was not adequate enough.

#### Patterns of adapting

##### Pattern I

People in this pattern attained optimal adequacy. These participants described knees that were symptom-free: “I don’t have any pain anymore.... it's like my original knee” and “I'm very satisfied. I don’t even notice it now.” People in Pattern I felt their new knee was supporting their life in the ways they had hoped and that it was allowing them to do the things they wanted to do. In the words of one interviewee, “When there’s no pain you’re able to do things that you wouldn’t have done if you were still in pain.”

Adapting was straightforward for people in Pattern I, and was inactive at the time of the study. People in this pattern did not dwell on aspects of either integrating or accepting, aside from mentioning that they had undertaken the standard course of physiotherapy which tended to be the only integrating activity described. Straightforwardness was implied by the small part these sub-processes played in their stories.

##### Pattern II

People in this pattern attained good adequacy. These participants described knees with lingering but manageable symptoms, for example: “I would say it’s better than [my old knee], so, satisfied—that’s the word.” In the words of another person in this group, “I would say seven out of ten. I no longer have the discomfort and the pain – and I just remember that being really bad.” While people in this pattern acknowledged minor persistent symptoms, they still felt their new knee was supporting their life in ways they had hoped. For instance, a participant described being able to move through life without fear of falling as an important trade-off with other symptoms: “Yes, I have the pain. I can get over that. Before my knee would give out. And I had fallen. And I don’t have that fear now.”

While adapting was inactive at the time of the study, this process was not entirely straightforward for people in Pattern II. The sub-process of integrating was slightly protracted—involving extra rehabilitation to try and improve the new knee, including additional physiotherapy treatment and consults with physicians. Efforts were also made to accommodate the new knee. For example, a participant with post-surgery limitations in range of motion and a passion for biking, worked with a bike store “to see about shortening the shank of the crankshaft, so then I would be able to get the full [rotation].” The sub-process of accepting involved more effort as well, including decision making about further integrating. At times, accepting involved reflection activities. In an instance of such reflection, one participant acknowledged their role in their knee falling a bit short of expectations, due to their lack of discipline in integrating: “... I cannot blame the doctor and I cannot blame the physio directly. I mean it was my responsibility to make sure that I did the exercises stringently and I – *oooh,* I’ll do this tomorrow.”

##### Pattern III

People in this pattern attained tolerable adequacy. These participants described persistent unresolved symptoms that were the source of ongoing disappointment and frustration. They were tolerating a new knee that was somewhat improved over their original knee, but remained distracting and debilitating. A participant described their new knee this way: “It’s better now than it was before. But I still have pain with it all the time, to varying degrees. Today it’s sore. Every day it’s sore to some degree. Some days are really quite sore.” These people’s stories revealed a disconnect between what they had expected and how their new knee felt and acted. These disconnects centred on unmet expectations, such as a worsening of the pain that prompted the person to have the surgery: “I understood that this surgery [would] alleviate the pain and unfortunately, in my case, it didn’t. On the contrary it seemed... the pain was more constant after the surgery.” People in this group described knees that prevented them from engaging in activities, including basic life activities: “I can't go out in the garden and bend. I have to bend from the waist because the knee won't bend, I can't get down on my knees.”

Although adapting was inactive for participants in Pattern III at the time of this study, adapting had not been straightforward for this group, requiring considerable extra effort. The sub-process of integrating was very protracted and involved additional therapies (e.g., extra physio, pool therapy) and interventions (e.g., manipulation), with minimal to no improvement. Efforts were also made to accommodate the new knee, such as hiring a housekeeper to assist with household chores or using medication to continue participation in a preferred recreational activity: “Yeah, so before I go [to the activity] and it’s the only time I take these... it’s Tramadol, and it kills the pain and the stiffness.” These efforts meant that the accepting sub-process involved ongoing decision making about how long one should keep trying to achieve further improvement. At the time of the study, this group had brought extra efforts at integrating to an end because they had given up hope that they would make a difference. In light of these frustrated efforts, many expressed disappointment about support from the health care system. There was a sense of being left too much on one’s own to figure out how to resolve persistent symptoms and of wanting “a little long-term follow-up.” In addition to decision making about bringing integrating to a close, accepting activities involved reflection on personal expectations as part of making peace with their new knee. These reflections included consideration of whether one’s original expectations had been reasonable: “I’m glad it’s improved over what it was. Am I satisfied? No. But maybe that’s because my expectations were too high.”

##### Pattern IV

People in this pattern had, to this point, attained insufficient adequacy. Participants in this group described ongoing, highly disruptive symptoms; for example, “I haven’t achieved pain removal, you know, I’ve still got it.” Continuing symptoms were the source of deep frustration and distress, often expressed in terms of unhappiness: “I am not happy with [the new knee]. I’ve never been happy with it. It’s just like a misery to me” and “I’ve been saying *from day one* [the new knee] has not been happy and that doesn’t make me happy.” People in this pattern described a substantial and, for many, intolerable gap between what they had hoped for from their TKA, and the reality of their new knee three to four years post-surgery. Someone went so far as to reflect that they might have been better off not having the surgery: “I wish that I hadn’t done it a lot of times. Because a lot of times when I’m in pain I wish to hell I’d not even thought of having this done.” People described knees that did not support their lives as hoped. Instead, their new knees added an ongoing burden: “If I ever want to walk or climb a step, it’s going to be a lot of hard work to get there. And I hate it.... because I can’t hardly walk anymore.”

Adapting was anything but straightforward for people in Pattern IV and was ongoing at the time of the study. The sub-process of integrating involved seeking both conventional and non-conventional interventions and multiple consults with surgeons, pain specialists, rehab providers, and family physicians, all to no significant avail. Efforts to accommodate the new knee included daily use of strong prescription medication and/or medical marijuana. Accommodations also involved adjustments to tasks, such as hanging on to kitchen counters and using stools as way-stations in order to cook. Another modification involved working around limitations getting down on the floor: “I’ve got to take a chair and hang onto it and slide down onto the floor. When I’m on the floor... I have to hang onto the edge of the desk and the leg, and pull myself up with the torso of my body because my leg just won’t let me push on it.” The sub-process of accepting was highly active, with multiple negative emotions—these participants were angry and frustrated at the state of their knee. People in this group were fully committed to seeking further help and searching for answers wherever they could find them. They described struggling with a health care system that they felt was letting them down. There were fears that the system might not hold the answer: “Am I going to be like this? I think I am because nobody’s going to help me. Nobody knows how.” There were also descriptions of feeling unheard: “And then I’d see the surgeon [who would say], ‘well, everything is the same as what’s in your other leg, and everything is where it’s supposed to be, so there you go’.” No one in this group was prepared to make peace with their knee as it was—all still sought a knee that fit better with their expectations. This resolve was aptly summed up by this participant: “There’s got to be something to do. I’m just not, I’m not prepared to lay down and die. I’ve got things to do yet.”

##### Pattern V

People in this pattern achieved tolerable adequacy. These participants described knees that had ongoing symptoms at the time of the study, but adapting had been pre-empted by emergent life events that occurred in close proximity to their surgeries, such as diagnoses with life-threatening conditions or the loss of a family member. Despite these ongoing symptoms, in the context of their lives and in light of more pressing competing demands, people in this group felt their knees were tolerable: “I’ve had to forge on in daily living, because life’s forced me to and you just tolerate the pain as you go.” These people acknowledged that their new knees were not necessarily supporting their lives as expected—but that, more importantly, their life situations were not as expected. These life situations took precedence over efforts to adjust to their new knee.

Given the priority of these life situations, adapting was inactive for people in Pattern V. To the extent adapting occurred before being pre-empted, it was described as straightforward and in fact played a very small role in these participants’ stories. These people did not detail aspects of either sub-process when telling their stories. Nonetheless, minimal integrating had taken place as all had undertaken basic rehab activities to the best of their abilities given the larger events in their lives. And their willingness to make do with less-than-perfect knees indicated that they had done some accepting work towards making sense of their new knees within the context of their lives. For example, one of these individuals reflected on their new knee’s relative importance in their life: “So you see, when I started looking at [multiple procedures and diagnostics related to an urgent health condition] and I've been more than 2 months in hospital in 3 years... when you start looking at my knee problem, I mean my knee problem's minor compared to that.”

### The influence of contextual conditions on adapting

Across all five patterns, there was evidence in participants’ stories that adapting was influenced by a variety of contextual conditions. The stories of people in Pattern V, for example, vividly demonstrated that other life events could disrupt adapting to the new knee. Participants described positive and negative effects of various personal characteristics on adapting—including their age, health status, personal support, location, and means. For instance, one participant acknowledged being “very very lucky” because they had good extended health insurance which enabled them to afford months of private physiotherapy and massage therapy, while another noted that accessing their rehab activities was facilitated by having family members to take them to appointments they would otherwise have trouble attending. Conversely, another participant felt their responsibility for taking care of their ailing spouse impeded their ability to complete their rehab sessions, while a rural participant had to travel for surgery, affecting both the timing of the procedure and the associated out-of-pocket costs. Within our sample, there were not enough data to map these influences into the five patterns, but it was clear that personal circumstances can play a modifying role in adapting, particularly with regard to integrating activities.

## Discussion

When asked about long-term satisfaction with their new knee three to four years post-TKA, our participants assessed their knee in terms of its perceived adequacy—how well their symptom resolution and regained function fit their expectations about how their new knee would feel and work. The use of grounded theory enabled us to identify the key process giving rise to these assessments of adequacy, namely the process of *adapting*—the experience of adjusting physically and cognitively to a new knee. While the idea of patients adapting following TKA surgery is not new [48], the adapting process has not previously been theorized as it is here. Significantly, for participants who did not readily achieve the adequacy they had anticipated, this process of adapting was the dominant thread in their stories. Their challenges integrating and accepting their new knee were keenly front of mind as they assessed their new knee.

While we approached this study anticipating that we might identify a new construct for patient satisfaction, we instead learned that what resonates for patients are concepts that are less all-encompassing and, at the same time, less summary in nature than satisfaction. While a level of “satisfaction” might be an efficient metric for clinicians and health policymakers, in the context of our study it held less meaning for our participants. Participants’ sense of the adequacy of their new knee, and their experience of adapting to achieve that adequacy, took primacy. The concept of adequacy and the adapting process, however, are closely tied to satisfaction. They are, in effect, satisfaction drivers. Given this relationship, these participants’ experiences adapting to their new knee afford us key insights into how the health care system could adjust to better support these patients, and improve rates of satisfaction with this high-volume procedure.

### Enhanced system support for physically integrating the post-TKA knee

When assessing the adequacy of their new knee, a key consideration was participants’ sense of how comfortably and well their new knee worked. This affirms the importance of known correlates of patient satisfaction such as symptom resolution and resumption of function [11, 13, 30, 31]. Negative associations have been reported between residual pain and compromised function and patients’ long-term satisfaction following TKA [49–51]. Shannak et al. [50], for example, looked at a sample of patients with a mean follow-up time of roughly 9 years and found that those who were dissatisfied reported pain, stiffness, and poor range of motion as the primary reasons for dissatisfaction. What our study contributes is a view into *how* these poorer outcomes are experienced and managed by patients as they adapt to their post-TKA knee. The stories of patients in Patterns III and IV, all of whom described notable shortfalls in the quality of their symptom resolution and function, detailed integrating experiences that were not straightforward. The more compromised their symptom resolution, the more their integrating activities relied on the health care system to support their efforts at improvement—including additional therapy, manipulations, pain management, and even a replacement of an original replacement. Participants whose additional efforts at integrating did not secure the results they wanted, expressed deep frustration with what they perceived to be a lack of adequate help from the health care system.

These less straightforward, less successful integrating experiences reveal a weakness in the current one-size-fits-all approach to post-TKA follow-up and rehabilitation services. Participants who took longer than anticipated to achieve adequate results spoke to an absence of readily available clinical support that extended beyond standard follow-up. This is a remediable service gap. For those with minor delays and setbacks in symptom resolution, this support could be managed through easier access to additional follow-up consults and greater flexibility in the number of prescribed and covered physiotherapy sessions. These would be a manageable system investment towards greater adequacy more quickly realized for the patient. For patients whose persistent symptoms are particularly tenacious, a more structured and extended clinical response could deliver major benefits to patients. This response could be managed through a care model that provides access to ongoing clinical and informational support and guidance navigating the system [38]. Such a model could be staffed by dedicated TKA nurse practitioners or patient navigators who are trained to assess complications and delays in TKA recovery and direct patients to the most appropriate system resources to address those issues [38, 52, 53]. Early evidence on orthopedic nurse navigator programs indicates that they can be cost-effective [54], reduce readmissions [52], and receive good patient feedback [55–57]. The approach would alleviate unnecessary and inappropriate excess demand on surgeons, and allow patients an easily accessible health system contact who could spend more time than a surgeon listening to and addressing their concerns.

### Enhanced system support for cognitively accepting the post-TKA knee

The other key input into assessments of adequacy was the extent to which the new knee met the expectations a person had going into the surgery. For those in our study whose symptoms did not resolve as anticipated, there was a range of negative responses that affected accepting their new knee—from surprise and disappointment that needed to be processed, to outright refusal to settle for a knee they felt was inadequate. The role of expectations has been studied relative to TKA patient satisfaction. There is evidence that TKA patients tend to have overly high expectations going into surgery [58–60]. While some studies have examined if pre-surgical expectations predict patient satisfaction [61–63], there is a growing recognition that the role of expectations may be better understood by looking at the effect of *fulfilment* of pre-surgical expectations on satisfaction [64–66]. Our findings affirmed the importance of fulfilment—the fit between pre-surgical expectations and post-surgical reality [64].

The importance of fulfilling pre-surgical expectations [64–66], and evidence that TKA patients may be carrying unrealistic expectations into these procedures [58–60], points to a key opportunity for the system to better support the accepting sub-process of adapting through a more consistent and systematic approach to working with patients to set appropriate informed expectations prior to surgery [58, 67]. While it was evident in our study that a few of the surgeons did talk with their patients about expectations, it was also evident that many did not. Further, while all our participants attended education sessions and/or received educational materials prior to surgery, these materials were not designed to shape expectations. Smith et al. [58] found that patients, despite exposure to comprehensive educational material, still tended towards unrealistic expectations about what the procedure would do, and concluded this could only be properly addressed in specific one-on-one discussions. Collaborating with patients to set expectations could be accomplished as a standard component of the pre-surgical consult. This discussion would include soliciting a patient’s views on what they hope for from the surgery and then modifying expectations as needed based on the clinician’s assessment of what the procedure could reasonably be expected to do for an individual patient [58, 59].

### Study limitations

Given our study’s cross-sectional design, the analysis was not able to shed light on how adapting unfolds immediately post-surgery. As it is reasonable to expect that the process of adapting would be most active in the first year following surgery, we anticipate that the most comprehensive understanding of how the sub-processes of integrating and accepting interact would come from data collected within that post-surgical time period. Exploring the concept of adapting in that pivotal first year would be a valuable addition to our understanding of the process, and could shed light on other opportunities to better support TKA patients.

Our study sample was limited in its diversity (e.g., ethnicity, income) as a consequence of our sampling frame, which was restricted to members of an existing patient cohort who expressed willingness to participate in further research. This may have resulted in a high level of homogeneity in the interviewees on certain characteristics. Future exploration into the nature of adapting would benefit from a more diverse sample.

Lastly, the nature of our interview guide questions did not render data that shed much light on the influence of personal contextual conditions. The experiences of participants in Pattern V, whose ability to adapt to their knee had been compromised by other life events, demonstrated that these personal conditions can have a considerable impact on a person’s experience adapting. A deeper exploration into other personal contextual conditions—such as financial means (e.g., ability to pay for extra treatment) or geographic location (e.g., rural system capacity)—could provide important insight into how contextual conditions facilitate or impede a person’s experience adapting.

## Conclusions

In our study, participants assessed their post-TKA knees in terms of its perceived adequacy. We found that the process of *adapting* to a post-TKA knee determines achieved adequacy. Both adequacy and the experience of adapting influenced our participants’ sense of satisfaction with their new knees. The process of adapting provides a number of opportunities for enhancing system support in ways that could improve long-term patient satisfaction. These opportunities include the development and evaluation of a care model that allows comprehensive long-term clinical support for patients whose knees do not achieve desired results on schedule, and collaborating with patients to set and manage reasonable expectations about how their new knee will feel and work following this procedure.

## Data Availability

The dataset generated and analyzed for this study is not publicly available because the study participants were not asked to provide permission to make their data available to other studies. This permission was not requested because, while protocols were rigorously followed to anonymize these qualitative interview data, there remains a risk that participants’ identities might be discernable given the nature of their stories.

## Abbreviations

BC: British Columbia
PEAK: Patient Experience with Knee Arthroplasty study
TKA: Total knee arthroplasty
